# Clinical Heterogeneity of Refractory Ascites After Pediatric Liver Transplantation Modulates Allograft Rejection Risk

**DOI:** 10.1002/mco2.71002

**Published:** 2026-09-11

**Authors:** Bingran Wang, Yunmu Gao, Jiaqi Song, Qi Pan, Aiwei Zhou, Hongyuan Liu, Wanglong Xiao, Zhicong Zhao, Taihua Yang, Chen Chen, Zhipeng Zong, Tao Zhou, Yi Luo, Yongbo Liu, Yuan Liu, Qiang Xia

**Affiliations:** ^1^ Department of Liver Surgery Renji Hospital Shanghai Jiao Tong University School of Medicine Shanghai People's Republic of China; ^2^ Shanghai Institute of Transplantation Renji Hospital Shanghai Jiao Tong University School of Medicine Shanghai People's Republic of China; ^3^ Shanghai Immune Therapy Institute Renji Hospital Shanghai Jiao Tong University School of Medicine Shanghai People's Republic of China; ^4^ Shanghai Engineering Research Center of Transplantation and Immunology Renji Hospital Shanghai Jiao Tong University School of Medicine Shanghai People's Republic of China

**Keywords:** allograft rejection, immune remodeling, pediatric liver transplantation, predictive model, refractory ascites

## Abstract

Refractory ascites (RA) is a severe complication that impedes long‐term survival after pediatric liver transplantation (LT), but its etiology remains complex and heterogeneous. To systematically evaluate the prognostic impact, clinical risk factors, and immunological features of RA, we retrospectively analyzed 1455 pediatric recipients who received LT in Renji Hospital between October 2016 and December 2021. RA was associated with increased mortality and graft loss after pediatric LT. Univariate analysis identified 13 clinical factors associated with RA, whereas a preliminary multivariable model showed only moderate discrimination, suggesting substantial heterogeneity among RA patients. Based on unsupervised clustering of clinical parameters, RA individuals were classified into two subgroups. Type I ascites was mainly associated with low graft‐to‐recipient weight ratio, decreased preoperative white blood cell count, and platelet count, indicating the involvement of persistent portal hypertension. In contrast, type II ascites was characterized by ABO incompatibility, elevated early postoperative soluble IL‐2 receptor, and increased risk of T‐cell‐mediated rejection, suggesting excessive alloimmune activation. These findings indicate that RA after pediatric LT comprises distinct clinical entities with different pathogenic implications. Prudent graft selection and close monitoring of immune status may help prevent RA and improve post‐transplant outcomes.

## Graphical abstract

Refractory ascites (RA) post‐pediatric LT is stratified into two subtypes: Type I (portal hypertension‐driven) and Type II (immune‐mediated). Type I is linked to low GRWR and hypersplenism, while Type II involves ABO incompatibility and early T‐cell activation (elevated sIL‐2R). Type II increases rejection risk (OR = 2.212). Precision management—graft selection for Type I and immune monitoring for Type II—can improve outcomes.

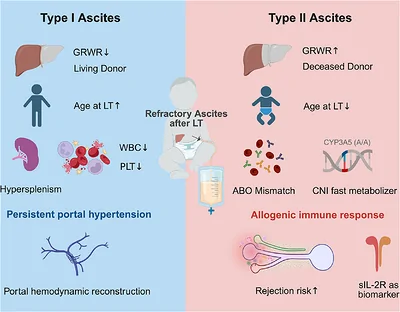

## 1 Introduction

Liver transplantation (LT) remains the only curative treatment for pediatric end‐stage liver disease (ESLD). During last decade, the post‐LT survival rates have greatly improved due to the advancements in perioperative management, surgical techniques, and immunosuppressor [1]. In spite of these achievements, there are still obstacles remain to be overcome for better prognosis. Refractory ascites (RA) was a common complication, which occurred in 5%–7% of adult LT recipients and was believed to be more common in living donor pediatric recipients. Our previous study has confirmed the contribution of RA to the poor prognosis after pediatric LT [2, 3]. As a result, understanding the pathogenesis of RA might improve the current management and prognosis of pediatric LT.

The etiology of RA after LT was poorly understood and currently believed to be related to outflow obstruction, infection, allogenic rejection, etc. [4, 5]. These mechanisms may not act independently, and different clinical backgrounds may contribute to different patterns of RA formation. Therefore, RA after pediatric LT should not be regarded as a single homogeneous complication, but rather as a clinical phenotype with mixed hemodynamic, metabolic, surgical, and immune‐related origins.

It has been revealed that graft type, ischemia time, ascites volume at laparotomy, and donor age are associated with the formation of RA [6, 7, 8], consistent with conventional perspectives about the involvement of portal hemodynamic disturbance in the pathogenesis of RA. Our previous studies identified that RA was closely related to alloimmune response and had an adverse effect on tolerance formation after pediatric LT, indicating the contribution of immune activation to RA [5].

Due to the above‐mentioned complexity of underlying mechanisms involved in RA formation, risk factors associated with RA still need further exploration, especially immune‐related factors. In addition, the contribution of many clinical parameters to the risk of RA would be nonlinear, and conventional logistic regression is inadequate to capture such complexity. More flexible modeling strategies that can accommodate nonlinear relationships are therefore required. Moreover, previous studies were limited due to the scale of cohort and focused mainly on adult recipients. There is still a lack of systematic studies with large‐scale samples on the prognostic impact and risk factors of ascites among pediatric LT recipients, where graft size, recipient age, and perioperative immune management may exert stronger effects.

In this retrospective cohort study, 1455 cases were analyzed to systematically reveal the effect of RA on post‐LT prognosis and identify predictors associated with RA. Further, restricted cubic spine (RCS) analysis was applied to identify the non‐linear contribution of each predictor to RA. We also explored the clinical heterogeneity of RA and identified two types of RA with different prognostic implications. Finally, a highly accurate and specific predictive model for RA was constructed and validated, serving as a useful clinical tool for decision‐making.

## 2 Results

### 2.1 RA Was Associated With Adverse Outcome After Pediatric LT

**FIGURE 1:**
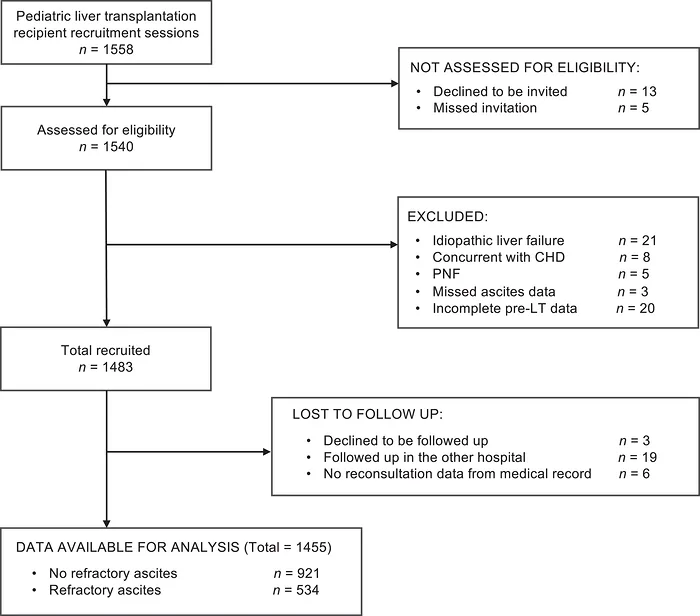
STROBE‐standardized participant flow diagram illustrating patient screening, exclusion, grouping, and sub‐cohort stratification in this study.

**FIGURE 2:**
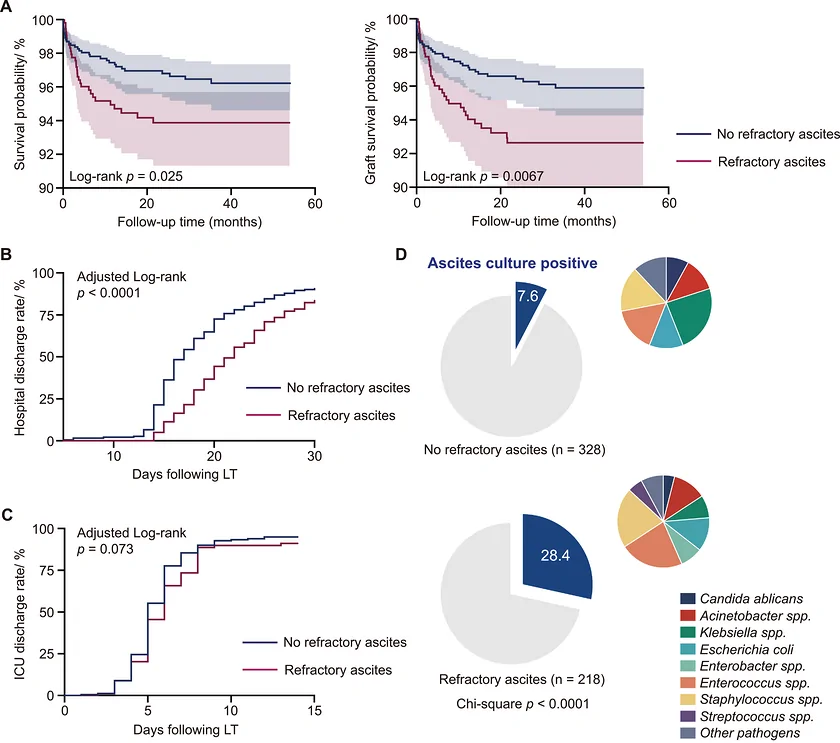
Comprehensive assessment of the contribution of RA to several adverse outcomes after pediatric LT. (A) Kaplan–Meier curves comparing the overall survival (left panel) and graft survival (right panel) between pediatric recipients who developed RA or not after LT. (B) Cumulative incidence curves for hospital discharge rate between recipients who developed RA versus those who did not. (C) Cumulative incidence curves for ICU discharge rate between recipients who developed RA versus those who did not. (D) Pie chart displaying the percentage and diversity of positive findings in ascites culture between two groups of pediatric LT recipients.

To comprehensively clarify the impact of RA on the long‐term survival after pediatric LT, we enrolled 1483 eligible pediatric recipients who received LT in our center. Among them, 28 patients (1.9%) were excluded from the final analysis due to incomplete follow‐up information (Figure 1), including patients who declined further follow‐up, received follow‐up care at another hospital, or had unavailable follow‐up records. To formally evaluate the potential impact of follow‐up attrition, we compared the incidence rate of RA between patients included in the final analysis and those excluded because of incomplete follow‐up. No significant differences were observed between these groups (OR = 0.669, 95% confidence intervals [CI]: 0.316–1.417, *p* = 0.294), suggesting that follow‐up attrition was unlikely to introduce substantial selection bias. Finally, we included 1455 pediatric LT recipients for retrospective analysis with a median follow‐up time of 2.6 years (Table 1). Kaplan–Meier analysis revealed a significantly increased mortality and graft loss in patients who developed RA after pediatric LT (Figure 2A), which was in agreement with our previous reports [9]. Further multivariant Cox regression also confirmed that RA was an independent risk factor for either post‐LT death (HR = 1.808, 95% CI: 1.098–2.976, *p* = 0.020) or graft loss (HR = 2.321, 95% CI: 1.549–3.358, *p* = 0.015) (Tables S1–S2). As expected, we also observed a prolonged hospital stay of recipients with RA (Figure 2B), while the duration of intensive care unit stay was comparable between two groups (Figure 2C). In addition, recipients who developed RA showed increased frequency and divergency of positive findings in ascites culture (Figure 2D), suggesting their susceptibility to peritoneal infection. Taken together, these data indicated that RA is a serious complication following LT and has significant impact on prognosis and quality of life.

**TABLE 1:** Baseline characteristics of participants enrolled in this study.

Variable	Refractory ascites	No refractory ascites	*p* value
*n*	534	921	
Age at LT (mo), median (IQR)	12.383 (6.5667, 45.833)	8.9 (6.2667, 22.867)	< 0.001
Types of transplantation, *n* (%)			0.050
SLT	62 (4.3%)	75 (5.2%)	
LDLT	396 (27.2%)	729 (50.1%)	
OLT	76 (5.2%)	117 (8%)	
Primary disease (category), *n* (%)			0.098
Cholestatic	410 (29.3%)	701 (50%)	
ALF	8 (0.6%)	16 (1.1%)	
Other	35 (2.5%)	32 (2.3%)	
Metabolic	47 (3.4%)	96 (6.9%)	
Tumor	21 (1.5%)	35 (2.5%)	
Height (cm), median (IQR)	72 (65.5, 97)	69 (64, 82)	< 0.001
Weight (kg), median (IQR)	8.8 (6.925, 15)	7.8 (6.5, 10.625)	< 0.001
Abdominal circumstances (cm), median (IQR)	50 (46, 56)	48 (44, 52)	< 0.001
Sex, *n* (%)			0.956
Male	252 (17.3%)	436 (30%)	0
Female	282 (19.4%)	485 (33.3%)	1

Abbreviations: ALF, acute liver failure; IQR, interquartile range; LDLT, living donor liver transplantation; LT, liver transplantation; OLT, orthotopic liver transplantation; SLT, split liver transplantation.

### 2.2 Decoding the Heterogeneity of RA

**FIGURE 3:**
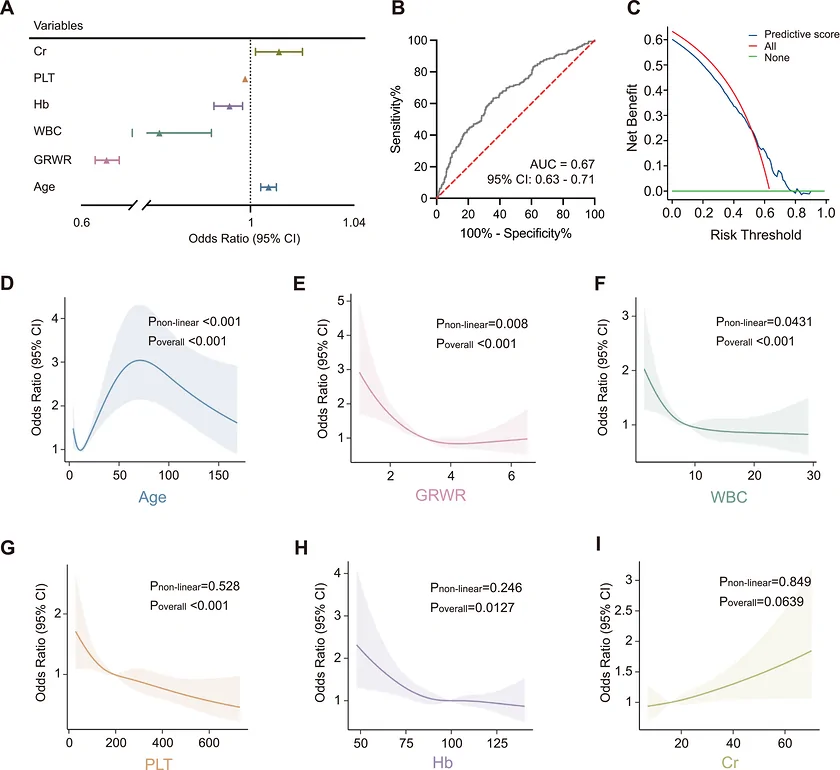
Performance of a preliminary predictive model of RA based on clinical parameters. (A) Forest plot displaying the univariate ORs with 95% CIs for the incidence of RA after pediatric liver transplantation. (B) ROC plot of the preliminary predictive model derived from multivariate logistic regression. (C) DCA curve of the preliminary predictive model for predicting RA formation. RCS plot visualizing the association of recipient age (D), GRWR (E), preoperative WBC (F), PLT (G), Hb (H), and Cr (I) with risk of RA. AUC, area under curve; Cr, creatinine; GRWR, graft to recipient weight ratio; HB, hemoglobin; PLT, platelet count; WBC, white blood cell count;.

Since RA was identified as a challenging problem, we next asked which clinical characteristics were related to the development of RA following LT. Univariate logistic regression analysis uncovered 13 factors that were associated with RA formation, including older recipients, low graft‐to‐recipient weight ratio (GRWR), ABO incompatibility, and decreased preoperative white blood cell count (WBC) (Figure 3A, Table 2). However, multivariate analysis only identified two independent risk factors of RA (Table 2), and the predictive model derived from logistic regression showed poor discrimination for patients with RA (AUC = 0.667, 95% CI (0.626–0.708), *p* < 0.0001) (Figure 3B), which contributed to moderate net benefits (Figure 3C). These findings indicated that specific clinical factors in different patient groups may have dual effects on the development of RA. As a result, we conducted RCS analysis to evaluate the dose–response relationship between each consecutive clinical parameter and RA. Linear association with RA risk was suggested for preoperative PLT, hemoglobin (Hb), and creatinine (Cr), while non‐linear associations were observed for age (*p* for non‐linearity <0.001) and GRWR (*p* for non‐linearity = 0.008) (Figure 3D–I). Regarding the strong U shape relation between recipient age and RA risk, we noticed a substantial reduction of RA risk within the lower range of age at LT, which reached the lowest risk around 12.5 months, either younger or older age increased RA risk significantly. It has been reported that small‐for‐size liver graft was a risk factor for RA with increased portal pressure, while increased GRWR would also lead to RA due to elevated intraabdominal pressure.

**TABLE 2:** Odds ratio of each clinical parameter to refractory ascites after pediatric liver transplantation.

Variable	Total (*N*)	Univariate analysis		Multivariate analysis	
		Odds ratio (95% CI)	*p* value	Odds ratio (95% CI)	*p* value
Age at LT, per month	1455	1.007 (1.004–1.010)	< 0.001		
Height, cm	1433	1.011 (1.006–1.015)	< 0.001		
Weight, kg	1438	1.028 (1.014–1.042)	< 0.001		
Abdominal circumstances, cm	782	1.040 (1.019–1.059)	< 0.001		
Primary disease	1401				
Cholestatic	1111	Reference		Reference	
ALF	24	0.855 (0.363–2.016)	0.720	0.825 (0.184–3.690)	0.801
Metabolic	143	0.837 (0.578–1.211)	0.346	0.365 (0.178–0.750)	0.006
Tumor	56	1.026 (0.589–1.786)	0.928	0.468 (0.193–1.134)	0.093
Other	67	1.869 (1.140–3.067)	0.013	1.001 (0.413–2.427)	0.998
Blood type	1455				
O	499	Reference			
A	426	1.114 (0.853–1.453)	0.429		
B	384	0.979 (0.743–1.292)	0.884		
AB	146	0.769 (0.518–1.143)	0.194		
ABO mismatch	1455				
Match	1299	Reference			
Incompatible	75	1.650 (1.034–2.632)	0.036		
Compatible	81	1.167 (0.737–1.848)	0.509		
Kasai	1455				
Yes	813	Reference			
No	642	1.292 (1.043–1.600)	0.019		
Graft weight, g	1437	1.000 (1.000–1.001)	0.243		
GRWR, %	1421	0.773 (0.696–0.858)	< 0.001	0.824 (0.680–0.999)	0.048
Recipient CYP3A5	1395				
*1/*3	577	Reference			
*3/*3	694	0.894 (0.711–1.126)	0.343		
*1/*1	124	1.618 (1.095–2.392)	0.016		
WBC	1439	0.965 (0.946–0.985)	< 0.001		
HB	1439	0.992 (0.986–0.997)	0.005		
PLT	1439	0.998 (0.997–0.999)	< 0.001		
ALB	1443	0.995 (0.985–1.005)	0.358		
TB	1443	1.001 (1.000–1.001)	0.127		
ALT	1443	1.000 (1.000–1.000)	0.804		
AST	1443	1.000 (1.000–1.000)	0.842		
Cr	1443	1.011 (1.002–1.020)	0.018		
NH3	1099	1.001 (0.998–1.003)	0.698		
Portal vein plasticity	1446				
No	1423	Reference			
Yes	23	1.114 (0.478–2.591)	0.804		
Portal stent	1447				
No	1433	Reference			
Yes	14	1.294 (0.446–3.745)	0.636		
Hepatic vein plasticity	1445				
No	1299	Reference			
Yes	146	1.316 (0.929–1.862)	0.122		
Number of hepatic arteries	1451				
1	1080	Reference			
2	363	1.040 (0.812–1.328)	0.761		
3	8	0.578 (0.116–2.882)	0.503		
Number of biliary tracts	1448				
2	156	Reference			
1	1285	0.951 (0.675–1.340)	0.775		
3	7	0.657 (0.124–3.496)	0.623		
Biliary anastomosis	1450				
Choledochojejunostomy	1230	Reference			
End‐to‐end anastomosis	220	1.102 (0.821–1.481)	0.515		
Roux‐en‐Y reconstruction	1448				
No	978	Reference			
Yes	470	1.212 (0.967–1.521)	0.096		
Intraoperative ascites	1455	1.001 (0.998–1.003)	0.619		

Abbreviations: ALB, albumin; ALF, acute liver failure; ALT, alanine aminotransferase; AST, aspartate aminotransferase; Cr, creatinine; GRWR, graft to recipient weight ratio; HB, hemoglobin; LT, liver transplantation; PLT, platelet count; TB, total bilirubin; WBC, white blood cell count.

**FIGURE 4:**
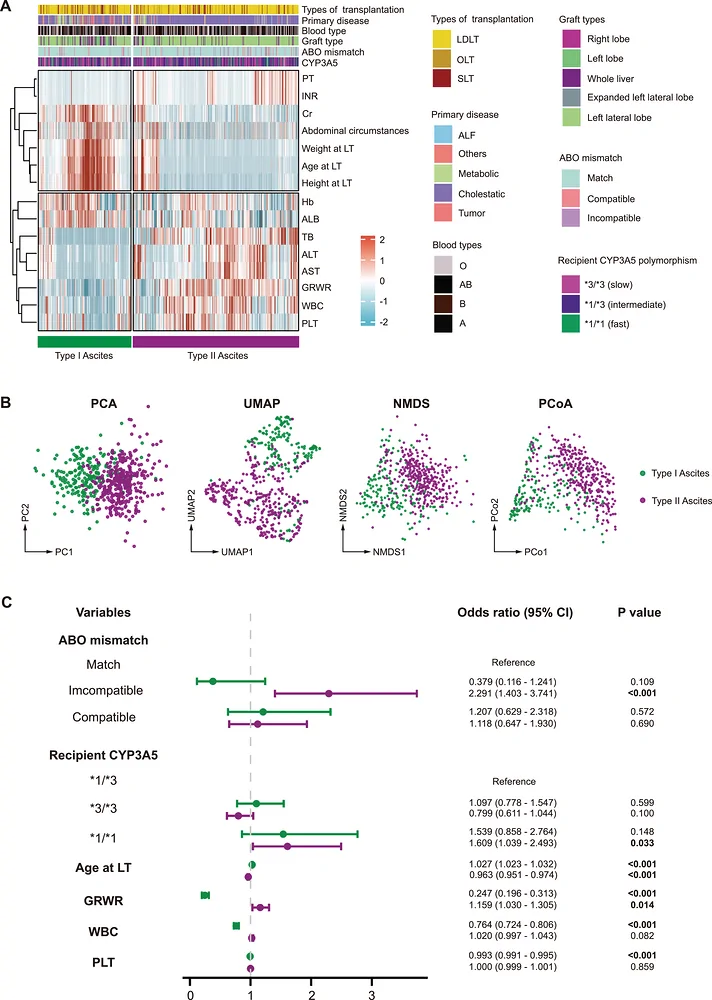
Identification of clinical heterogeneity of RA. (A) Heatmap displaying relative values of each clinical parameters and the clustering of pediatric recipients who developed RA into two based on Canberra distance. (B) Dot plots showing the distribution of identified two groups of ascites under four unsupervised clustering algorithms: PCA, UMAP, NMDS, and PCoA. (C) Forest plot comparing the contribution of indicated clinical parameter to each group of ascites. ALB, albumin; ALF, acute liver failure; ALT, alanine aminotransferase; AST, aspartate aminotransferase; Cr, creatinine; GRWR, graft to recipient weight ratio; Hb, hemoglobin; INR, international normalized ratio; LDLT, living donor liver transplantation; LT, liver transplantation; OLT, orthotopic liver transplantation; PLT, platelet count; PT, prothrombin time; SLT, split liver transplantation; TB, total bilirubin; WBC, white blood cell count.

Due to the non‐linear relationship between clinical factors and RA formation, it is necessary to apply subgroup analysis on RA. To understand the impact of each clinical factor on RA formation, we need to decode the heterogeneity of RA. Based on the Canberra distance of clinical parameters identified above, we classified RA individuals into two clusters (Figure 4A), termed type I ascites and type II ascites. This pattern was replicated by other unsupervised clustering algorithms, including PCA, UMAP, NMDS, and PoCA (Figure 4B). As expected, age at LT and GRWR showed opposite contributions to RA formation in different groups (Figure 4C), consistent with results from RCS analysis. Multivariant logistic regression for each type of ascites revealed that low GRWR, decreased preoperative WBC, and PLT were independent risk factors for type I ascites (Table 3), indicating the involvement of persistent portal hypertension. Notably, ABO incompatibility between donor and recipients was confirmed as the independent risk factor for type II ascites (Table 4). In addition, CYP3A5 single nucleotide polymorphism affected the pharmacokinetics of calcineurin inhibitor (CNI) and our previous reports revealed its connection with spontaneous immune tolerance in pediatric LT recipients [5]. Here, we observed more CYP3A5 *1/*1 carriers (fast metabolism) in type II ascites (Figure 4C), further confirming the involvement of relative IS insufficiency and immune activation in the pathogenesis of type II ascites, which was rarely discussed in previous research. Collectively, these data revealed that type I ascites was previously recognized as RA due to small‐for‐size liver graft. To elucidate the optimal range of GRWR, within which recipients experienced lowest RA risk, we applied RCS analysis for these two subtypes of RA. We observed a significantly increased risk for type I ascites when GRWR was less than 2%, while GRWR did not contribute to increased RA risk in type II ascites (Figure S1A). Further threshold analysis confirmed that GRWR less than 1.8% was the precise knot of type I ascites.

**TABLE 3:** Clinical factors associated with type I ascites after pediatric liver transplantation.

Variable	Total (*N*)	Univariate analysis		Multivariate analysis	
		Odds Ratio (95% CI)	*p* value	Odds Ratio (95% CI)	*p* value
Age at LT, per month	1105	1.027 (1.023–1.032)	< 0.001	1.008 (0.987–1.030)	0.457
Height, cm	1088	1.048 (1.041–1.055)	< 0.001	1.045 (1.001–1.091)	0.044
Weight, kg	1091	1.111 (1.089–1.133)	< 0.001	0.858 (0.786–0.938)	< 0.001
Abdominal circumstances, cm	596	1.150 (1.116–1.186)	< 0.001	1.051 (0.987–1.118)	0.119
Primary disease	1401				
Cholestatic	804	Reference		Reference	
ALF	21	2.127 (0.763–5.929)	0.149	7.159 (1.041–49.245)	0.045
Metabolic	126	2.127 (1.344–3.366)	0.001	1.920 (0.605–6.094)	0.268
Tumor	53	3.500 (1.911–6.409)	< 0.001	1.609 (0.474–5.464)	0.445
Other	60	5.955 (3.444–10.298)	< 0.001	1.947 (0.561–6.757)	0.294
Blood type	1105				
A	310	Reference			
O	376	0.964 (0.641–1.450)	0.861		
B	301	1.161 (0.764–1.763)	0.484		
AB	118	0.855 (0.471–1.550)	0.605		
ABO mismatch	1105				
Match	1002	Reference			
Incompatible	42	0.379 (0.116–1.241)	0.109		
Compatible	61	1.207 (0.629–2.318)	0.572		
Kasai	1105				
No	483	Reference		Reference	
Yes	622	0.630 (0.459–0.866)	0.004	1.898 (0.765–4.710)	0.167
GRWR, %	1076	0.247 (0.196–0.313)	< 0.001	0.316 (0.197–0.509)	< 0.001
Recipient CYP3A5	1053				
*1/*3	428	Reference			
*3/*3	544	1.097 (0.778–1.547)	0.599		
*1/*1	81	1.539 (0.858–2.764)	0.148		
WBC	1090	0.764 (0.724–0.806)	< 0.001	0.909 (0.821–1.006)	0.066
HB	1090	0.998 (0.990–1.006)	0.638		
PLT	1090	0.993 (0.991–0.995)	< 0.001	0.997 (0.994–1.000)	0.051
ALB	1094	1.003 (0.996–1.010)	0.344		
TB	1094	0.994 (0.992–0.996)	< 0.001	1.000 (0.997–1.003)	0.955
ALT	1094	0.996 (0.994–0.997)	< 0.001	0.998 (0.993–1.003)	0.464
AST	1094	0.997 (0.996–0.998)	< 0.001	0.999 (0.995–1.003)	0.544
Cr	1094	1.064 (1.048–1.081)	< 0.001	0.993 (0.955–1.032)	0.728

Abbreviations: ALB, albumin; ALF, acute liver failure; ALT, alanine aminotransferase; AST, aspartate aminotransferase, Cr, creatinine; GRWR, graft to recipient weight ratio; HB, hemoglobin; LT, liver transplantation; PLT, platelet count; TB, total bilirubin; WBC, white blood cell count.

**TABLE 4:** Clinical factors associated with type II ascites after pediatric liver transplantation.

Variable	Total (*N*)	Univariate analysis			
		Odds ratio (95% CI)	*p* value	Odds ratio (95% CI)	*p* value
Age at LT, per month	1,260	0.963 (0.951–0.974)	< 0.001	0.978 (0.939–1.017)	0.266
Height, cm	1,241	0.963 (0.952–0.974)	< 0.001	1.044 (0.977–1.116)	0.206
Weight, kg	1,245	0.875 (0.837–0.915)	< 0.001	0.830 (0.645–1.069)	0.149
Abdominal circumstances, cm	663	0.951 (0.924–0.980)	< 0.001	1.004 (0.953–1.057)	0.893
Primary disease	1207				
Cholestatic	1003	Reference		Reference	
ALF	18	0.290 (0.066–1.270)	0.100	2.040 (0.157–26.530)	0.586
Metabolic	112	0.387 (0.224–0.668)	< 0.001	0.849 (0.332–2.170)	0.732
Tumor	36	0.066 (0.009–0.486)	0.008	0.316 (0.038–2.600)	0.284
Other	38	0.435 (0.180–1.052)	0.065	1.411 (0.342–5.828)	0.634
Blood type	1260				
A	369	Reference		Reference	
O	439	0.916 (0.675–1.244)	0.575	0.943 (0.585–1.521)	0.811
B	324	0.759 (0.542–1.064)	0.110	0.692 (0.405–1.183)	0.178
AB	128	0.629 (0.390–1.017)	0.059	0.899 (0.443–1.827)	0.769
ABO mismatch	1260				
Match	1122	Reference		Reference	
Incompatible	70	2.291 (1.403–3.741)	< 0.001	2.042 (1.014–4.109)	0.045
Compatible	68	1.118 (0.647–1.930)	0.690	1.294 (0.575–2.911)	0.534
Kasai	1260				
No	536	Reference			
Yes	724	0.894 (0.696–1.149)	0.383		
GRWR, %	1233	1.159 (1.030–1.305)	0.014	0.918 (0.738–1.143)	0.446
Recipient CYP3A5	1209				
*1/*3	507	Reference		Reference	
*3/*3	598	0.799 (0.611–1.044)	0.100	0.852 (0.565–1.286)	0.446
*1/*1	104	1.609 (1.039–2.493)	0.033	1.701 (0.885–3.271)	0.111
WBC	1245	1.020 (0.997–1.043)	0.082	1.008 (0.970–1.048)	0.670
HB	1245	0.988 (0.982–0.995)	< 0.001	0.987 (0.975–0.999)	0.032
PLT	1245	1.000 (0.999–1.001)	0.859		
ALB	1249	0.972 (0.954–0.991)	0.004	0.994 (0.967–1.022)	0.687
TB	1249	1.003 (1.002–1.004)	< 0.001	1.002 (1.000–1.003)	0.016
ALT	1249	1.000 (1.000–1.001)	0.687		
AST	1249	1.000 (1.000–1.001)	0.135		
Cr	1249	0.939 (0.918–0.960)	< 0.001	0.970 (0.934–1.006)	0.105

Abbreviations: ALB, albumin; ALF, acute liver failure; ALT, alanine aminotransferase; AST, aspartate aminotransferase; Cr, creatinine; GRWR, graft to recipient weight ratio; HB, hemoglobin; LT, liver transplantation; PLT, platelet count; TB, total bilirubin; WBC, white blood cell count.

### 2.3 Shared and Varied Impact of Two RA Groups on Clinical Outcomes

**FIGURE 5:**
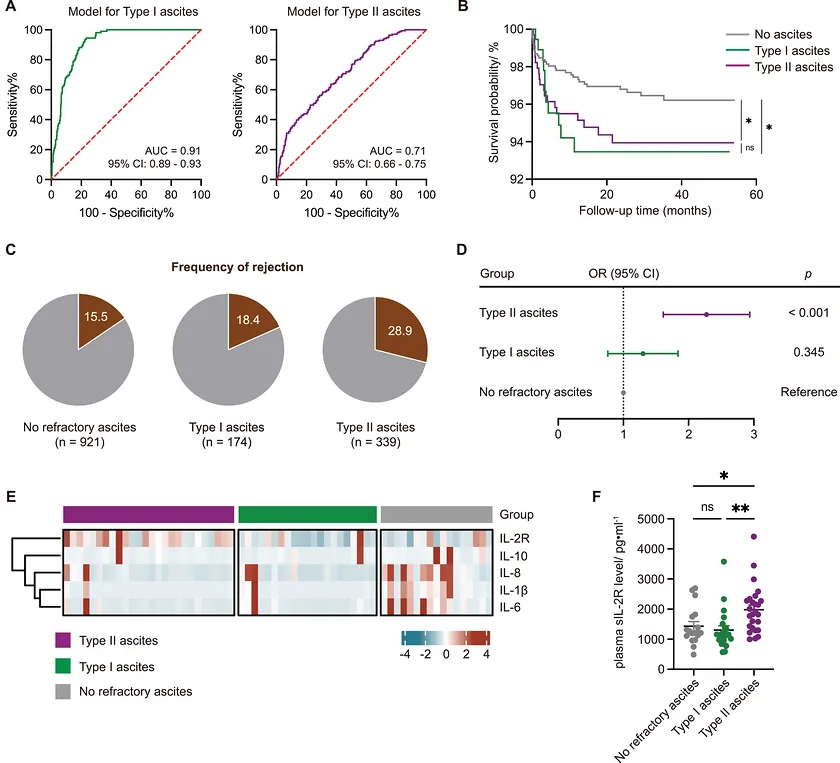
Construction of predictive models for two types of ascites and assessment of their association with allograft rejection risk. (A) ROC plots of the predictive models for type I (left panel) and type II ascites (right panel) derived from multivariate Logistic regression. (B) Kaplan–Meier curves comparing the overall survival among recipients who did not develop RA and those who developed type I and type II ascites after LT. (C) Pie chart comparing the incidence of allogenic rejection across indicated groups of recipients. (D) Forest plot visualizing the risks of rejection in different group of ascites. (E) Heatmap displaying the relative values of indicated cytokines across three groups of LT recipients. (F) Scatter plots showing the plasma concentration of sIL‐2R from three groups of LT recipients: No refractory ascites (*n* = 17), Type I ascites (*n* = 21), and Type II ascites (*n* = 26). AUC, area under curve.

Since we identified RA as complications with heterogenous originality, we established predictive models for two groups of RA separately. These two models showed favorable discrimination for type I ascites (AUC = 0.91, 95% CI [0.89–0.93], *p* < 0.0001) and type II ascites (AUC = 0.71, 95% CI [0.66–0.75], *p* < 0.0001), respectively (Figure 5A). DCA also confirmed the great net benefit gained by using these two models to predict RA after LT (Figure S1B). Calibration curves demonstrate good agreement between predicted and observed probabilities in each model (Hosmer–Lemeshow test, Type I: *χ* ^2^  = 11.3, *p* = 0.185; Type II: *χ* ^2^  = 7.45, *p* = 0.489, Figure S1C). Brier score of models for type I ascites (Brier score = 0.097, 95% CI: 0.083–0.114) and type II ascites (Brier score = 0.182, 95% CI: 0.166–0.199) also indicated favorable overall performance. Next, we asked whether our grouping have relevance to clinical outcomes. As expected, both type I and II ascites affected long‐term survival after pediatric LT, while there was no significant difference between these two types of ascites (Figure 5B). Notably, we observed an increased incidence rate of T‐cell‐mediated rejection (TCMR) in patients who developed type II ascites (Figure 5C). Logistic regression analysis also validated the contribution of type II ascites to increased TCMR risk (OR = 2.212, 95% CI [1.647–2.967], *p* < 0.001) (Figure 5D). Taken together, these results further indicated the involvement of allogenic immune response in type II ascites.

To further investigate the immune status of type I and type II ascites, we conducted a cytokine profile quantification assay on plasma from pediatric recipients 5 days after LT (Figure 5E). We noticed a significant elevation of plasma soluble IL‐2 receptor (sIL‐2R) in patients who developed type II ascites compared with those who developed type I ascites or did not develop ascites (Figure 5F). IL‐2R is a crucial receptor expressed on T cells upon activation, where it transduces signals that promote T‐cell survival and proliferation. Meanwhile, IL‐2R is also released into the circulation in its soluble form, known as soluble IL‐2R (sIL‐2R). Elevated plasma sIL‐2R was proven to be related to several inflammatory conditions, including sepsis, multiple sclerosis, and leukemia. Consequently, these results confirmed that increased allogenic rejection risk in type II ascites was related with early T‐cell activation following LT.

## 3 Discussion

In this large retrospective cohort of 1455 pediatric LT recipients, we confirmed that RA is a clinically meaningful complication associated with impaired overall survival, graft survival, and prolonged hospitalization. More importantly, our data show that RA after pediatric LT is heterogeneous. Unsupervised clustering separated RA into two clinically distinct subgroups: type I ascites, which was characterized by low GRWR, decreased preoperative white blood cell and platelet counts, and features compatible with persistent portal hypertension, and type II ascites, which was enriched in ABO‐incompatible transplantation, CYP3A5 fast‐metabolizer status, early postoperative sIL‐2R elevation, and increased TCMR risk. These findings provide the conceptual basis for interpreting RA as a syndrome arising from different combinations of graft‐recipient size matching, portal hemodynamics, and alloimmune activation, rather than as a single postoperative complication.

Previous studies have established persistent or RA as an adverse event after LT and have linked it to impaired hepatic venous outflow, portal hypertension, infection, hypoalbuminemia, and rejection [3, 4, 5, 10]. Studies in adult living donor LT further identified several factors, including graft type, ischemia time, ascites volume at laparotomy, donor age, and low GRWR, as contributors to post‐transplant ascites [6, 7, 8]. Our results are consistent with these observations in showing that graft size and portal‐hypertensive burden remain central determinants of RA. However, the present study differs from previous work in three important aspects. First, it focuses on a large pediatric cohort, in which recipient age, living donor graft selection, and perioperative immune management may have stronger effects than in adult cohorts. Second, rather than treating RA as a homogeneous endpoint, we applied non‐linear modeling and unsupervised clustering to uncover clinically interpretable subtypes. Third, we identified an immune‐related RA subgroup associated with early T‐cell activation and subsequent TCMR, a dimension that has rarely been systematically characterized in pediatric LT.

The literature on graft size may explain why RA is poorly captured by a single endpoint. The international consensus on small‐for‐size syndrome treats GRWR as a screening measure, not a physiological diagnosis, because the consequences of graft size depend on portal inflow per unit graft mass, venous outflow, portosystemic shunting, recipient disease severity, and graft quality [11]. At the other end of the range, pediatric recipients can tolerate a very high GRWR with excellent graft and patient survival if abdominal domain and vascular geometry are managed, although delayed abdominal closure is more common [12]. A study using GRWR greater than 4% to define large‐for‐size grafts documented perioperative hemodynamic and metabolic differences [13]. By contrast, registry data for pediatric whole‐organ transplantation implicated absolute donor weight, rather than the donor‐recipient weight ratio, as the main predictor of early graft loss [14]. These findings are not necessarily contradictory. They fit the U‐shaped, subtype‐specific pattern in our cohort, in which low and high GRWR may reflect different biological problems and the association varies with graft type and recipient age.

The type I subgroup largely corresponds to the mechanism most emphasized in earlier studies: persistent portal hypertension after implantation of a relatively small graft. The association of type I ascites with low GRWR, older recipient age, and low preoperative WBC and platelet counts supports a pathophysiological pathway in which pre‐existing portal hypertension and hypersplenism occur after LT when the graft is insufficient to accommodate portal inflow. This interpretation is further supported by previous reports that selected patients with RA after LT can benefit from splenic artery embolization, which reduces portal inflow and alleviates ascites [15, 16, 17]. Moreover, our study refines this traditional explanation by identifying a quantitative risk window in pediatric recipients. GRWR below approximately 1.8%–2.0% was specifically linked to type I ascites, whereas the lowest overall RA risk occurred within an intermediate GRWR range. These findings suggest that GRWR should be interpreted together with recipient age and preoperative portal‐hypertensive burden rather than by graft size alone.

The clinical implications of this portal‐hemodynamic mechanism are most relevant to selected patients with type I ascites. In one cohort with post‐transplant portal hyperperfusion, proximal splenic artery embolization lowered portal velocity and wedged hepatic venous pressure, with resolution of RA [18]. A systematic review found that splenic artery ligation or embolization, splenectomy, and portosystemic shunting can reduce portal hyperperfusion; however, intervention thresholds and the choice of procedure differed across centers [19]. Our 1.8% knot should therefore prompt closer hemodynamic evaluation, not intervention by itself. Decisions about inflow modulation need to account for Doppler findings, hepatic venous outflow, splenic size, collateral circulation, and graft function.

By contrast, type II ascites appears to represent a different biological entity. In this study, type II ascites was associated with ABO incompatibility, CYP3A5 *1/*1 fast‐metabolizer status, elevated early postoperative sIL‐2R, and increased TCMR risk. These features suggest that type II ascites may arise from early alloimmune activation under conditions of relatively insufficient immunosuppression or heightened graft immunogenicity. The association with CYP3A5 fast metabolism is biologically plausible because faster tacrolimus clearance may reduce effective CNI exposure during the critical early post‐transplant period [20]. ABO‐incompatible transplantation may further amplify endothelial and humoral immune activation, while early T‐cell activation, reflected by sIL‐2R elevation, may promote graft inflammation, sinusoidal endothelial injury, and delayed restoration of portal‐sinusoidal homeostasis. The observed association between high GRWR and type II ascites may also be explained by large‐for‐size physiology, in which graft crowding, impaired perfusion, and ischemia‐reperfusion injury could intensify inflammatory and alloimmune responses.

CYP3A5 provides a plausible link between type II ascites and early tacrolimus exposure. In pediatric living donor LT, expression of CYP3A5*1 in the donor graft was associated with more rapid tacrolimus clearance early after transplantation [21]. A separate pediatric population pharmacokinetic analysis found that apparent clearance also varied with body weight, postoperative time, and CYP3A5*1 carriage [22]. Genotype alone, however, does not establish inadequate immunosuppression. Testing this pathway will require longitudinal trough concentrations, dose‐normalized exposure, concomitant medications, liver function, and adherence data. The sIL‐2R result calls for the same caution. In a prospective liver‐transplant study, circulating sIL‐2R and CD25 expression increased during biopsy‐proven acute rejection [23], but infection and systemic inflammation can produce similar elevations. In this cohort, sIL‐2R is informative because its early increase coincided with a later excess of TCMR; it should not be used as an independent diagnostic marker.

Evidence on ABO‐incompatible LT is not uniformly adverse and depends heavily on the treatment context. A pediatric living donor series using rituximab‐based desensitization found similar rates of acute cellular rejection and other major complications in ABO‐incompatible and compatible recipients, although some ABO‐incompatible recipients still developed antibody‐mediated rejection [24]. In a meta‐analysis, ABO incompatibility was associated overall with more antibody‐mediated rejection and poorer graft survival, but the differences were smaller in pediatric recipients and those treated with rituximab [25]. Earlier pediatric experience also reported acceptable survival with standard immunosuppression and selective postoperative plasmapheresis [26]. Thus, in our clustering model, ABO incompatibility is better interpreted as one component of immunological stress. Its effect may depend on age, isohemagglutinin titers, desensitization, and calcineurin‐inhibitor exposure, rather than determining type II ascites on its own.

Because TCMR, antibody‐mediated injury, and nonspecific postoperative inflammation can present together, the immune phenotype requires confirmatory testing. The 2016 Banff update distinguished TCMR from antibody‐mediated rejection and incorporated donor‐specific antibodies and C4d into the assessment of humoral injury [27]. In pediatric LT, class II donor‐specific antibodies have been associated with progressive graft fibrosis on protocol biopsy [28]. DQ‐directed or complement‐binding donor‐specific antibodies have also been linked to late allograft dysfunction and rejection phenotypes [29]. For patients with type II ascites, persistent immune activation or abnormal liver tests should therefore prompt donor‐specific antibody testing and consideration of biopsy. Ascites or a single sIL‐2R value is not sufficient reason to intensify immunosuppression. Vascular, infectious, and mechanical causes should first be excluded; tacrolimus exposure, donor‐specific antibodies, serial immune markers, and histology can then be used to determine whether alloimmune injury is present.

RA should not be regarded only as a manifestation of persistent portal hypertension or surgical outflow disturbance; in a subset of pediatric recipients, it may be an early clinical signal of immune remodeling and rejection susceptibility. Practically, the subtype‐based framework may improve perioperative risk stratification. For patients at risk of type I ascites, attention should be paid to graft size selection, preoperative portal‐hypertensive status, and postoperative strategies aimed at remodeling portal hemodynamics. For patients at risk of type II ascites, closer monitoring of tacrolimus exposure, sIL‐2R, donor‐specific antibodies, and immune phenotypes may be warranted, together with early assessment for rejection when ascites is accompanied by biochemical or immunological abnormalities. Such a stratified approach is more clinically useful than applying a single management strategy to all RA patients.

The difference in model performance is also consistent with the proposed subtype mechanisms. Type I ascites, which is defined largely by measurable graft‐recipient size and portal‐hypertensive variables, was predicted with high discrimination (AUC 0.91). The type II model performed more modestly (AUC 0.71), possibly because drug exposure and immune activation vary over time and are not fully captured at baseline. Baseline data may therefore be adequate for initial hemodynamic risk stratification but less informative for immune‐related ascites, where serial postoperative measurements could improve prediction. External studies should test discrimination, calibration, and cluster reproducibility, as well as the stability of subtype assignment over time and the effect of subtype‐guided management on patient outcomes.

Several limitations of this study should also be acknowledged. First, this was a single‐center retrospective study, and although the sample size was large and internal bootstrap validation was performed, external validation in independent multicenter pediatric cohorts is required before the predictive models and GRWR thresholds can be generalized. Second, the clustering strategy was based on available clinical variables; therefore, the identified subtypes should be considered clinically useful phenotypes rather than definitive mechanistic categories. Third, the immune analysis was limited to a targeted cytokine panel at postoperative day 5 and was performed in a subset of recipients, which restricts the ability to reconstruct dynamic immune remodeling. Fourth, direct mechanistic evidence from paired ascites fluid, graft biopsy, portal hemodynamic measurements, and longitudinal tacrolimus exposure was not available for all patients. Future studies should prospectively combine serial clinical data, pharmacokinetic monitoring, donor‐specific antibody testing, flow cytometry or single‐cell profiling, ascites‐fluid proteomics, and graft transcriptomics to validate the biological basis of type I and type II ascites. Interventional studies are also needed to test whether subtype‐guided strategies, such as portal inflow modulation for type I ascites and individualized immunosuppression for type II ascites, can reduce RA incidence and improve long‐term graft outcomes. Fifth, even though the effect of loss‐to‐follow‐up was evaluated, we cannot completely exclude the possibility that unmeasured differences among patients with incomplete follow‐up, such as disease severity or healthcare accessibility, may have influenced long‐term outcomes. Therefore, the association between RA and adverse clinical outcomes should be interpreted with consideration of the potential residual confounding caused by incomplete follow‐up.

In summary, this study demonstrates that RA after pediatric LT comprises at least two clinically and biologically distinct entities. Type I ascites is mainly linked to persistent portal hypertension and low GRWR, whereas type II ascites is associated with early alloimmune activation and increased rejection risk. By distinguishing these subtypes, our findings refine the interpretation of RA, explain why previous studies identified heterogeneous risk factors, and provide a basis for more precise prevention and management of post‐transplant ascites in pediatric LT recipients.

## 4 Materials and Methods

### 4.1 Study Design and Participants

This single‐center cohort study aims to investigate the impact of RA on the prognosis of pediatric LT and to identify potential risk factors, with a particular focus on immune‐related mechanisms. The study was conducted in Renji Hospital, affiliated with Shanghai Jiao Tong University School of Medicine. From October 2016 to December 2021, a total of 1455 pediatric LT recipients were included in the study. We applied a self‐developed follow‐up system to collect clinical data of pediatric LT recipients, including laboratory results, medical history, daily immunosuppressants (IS) dosages, complications, treatments, and developmental information. Among all recipients, 1455 patients with intact follow‐up records were involved in this study. All transplants were performed in recipients younger than 18 years of age. Written informed consent was obtained from all participants or their legal guardians before inclusion in this study. The study followed the guidelines of the Declaration of Helsinki and was approved by the Institutional Review Boards (IRBs) of Renji Hospital (KY2022‐117‐B).

### 4.2 Definition of RA and TCMR

RA was defined as daily abdominal drainage exceeding 200 mL persistent for over 10 days after transplantation and could not be satisfactorily treated by medical therapy. Bacteria culture for abdominal drainage would be conducted to identify pathogenic microorganisms when infection was suspected. We recommended protocol liver biopsy for pediatric recipients every 1 year after LT. Diagnostic liver biopsy was indicated when liver enzyme elevated. TCMR was identified by histological findings according to Banff schema [30].

### 4.3 Statistical Analysis

We applied R package “survival [3.3.1], ggplot2 [3.3.6]” to make and visualize Kaplan–Meier analysis and log‐rank tests. Hazard ratios (HR) and 95% CI were also computed and results were formatted for clarity. The ROC were analyzed by R package “pROC [1.18.0],” and visualized by R package “ggplot2 [3.3.6].” Univariate logistic regression analysis was analyzed by R package “rms [6.4.0].” Further multivariant logistic regression model was established with the help of glm function. The *χ* ^2^ test and Wald test were applied to estimate the differences for categorical and continuous variables in logistic regression analysis, respectively. DCA curve was visualized with “rmda[1.6].” Hosmer–Lemeshow goodness‐of‐fit test was performed to evaluate calibration plots. RCS analysis was conducted by rms [6.4.0]. Both the overall association and the nonlinearity were evaluated: overall *p*‐values were derived from the model fit, while the Wald test was used to specifically assess the significance of the nonlinear trend. UMAP, NMDS, and PoCA were performed using the R packages: umap [0.2.10.0], vegan [2.6.4], and ape [5.6.2]. Dimensionality reduction results were visualized with “ggplot2 [3.3.6],” and clustering significance was assessed where applicable. The R package “ComplexHeatmap [2.13.1]” was used to create heatmaps. Clustering was applied where necessary to reveal patterns across samples and features. One‐way ANOVA was performed using GraphPad Prism to compare group means. Tukey's post hoc test was applied for multiple comparisons. All statistical tests were two‐tailed. *p*‐value less than 0.05 was considered statistically significant. All analyses were performed on open‐source software R version 4.2.1 and GraphPad Prism version 9.5.1.

## Author Contributions

B.W., Y.G., Q.P., and A.Z. conducted data collection and primary analysis. B.W., Y.G., J.S., and W.X. conducted deep data analysis and article draft. H.L., Z. Zhao, T.Y., C.C., Z. Zong, T.Z., Y. Luo, and Q.X. conducted most of the LT operations and are responsible for long‐term follow‐up management. Yuan Liu, Yongbo Liu, and Q.X. participated in article revision and supervision. All authors have read and approved the final manuscript.

## Funding

This study was supported by the National Natural Science Foundation of China (82471804, 825B2017, 82588101, 82371791), Shanghai Top Priority Research Center Class A (2022ZZ01016), Shanghai Science and Technology Commission (22Y21900400, 24QA2704900), Shanghai Municipal Health Commission (20244Y0134), and Shanghai Immune Therapy Institute.

## Ethics Statement

This research was approved by the Institutional Review Board (IRB) of Renji Hospital Ethical Committee (KY2022‐117‐B). Written informed consent was obtained from all participants and/or their legal guardians before inclusion in this study. The consent process involved a detailed explanation of the study objectives, procedures, potential risks, and data usage. For children aged ≤ 8 years, consent was obtained from their legal guardians after an age‐appropriate explanation was provided. For children older than 8 years, both participant assent and written informed consent from their legal guardians were obtained. The legal guardianship relationship was verified according to local regulatory requirements.

## Conflicts of Interest

The authors declare no conflicts of interest.

## Supporting information




**Supporting File 1**: mco271002‐sup‐0001‐SuppMat.docx

## Data Availability

Data will be shared with bona fide researchers who submit a research proposal approved by the independent review board. Individual recipient data will be shared in data sets in a de‐identified and anonymized format. Any researchers requiring original data could contact the corresponding author.
