# Supplementary material for: Clinical Heterogeneity of Refractory Ascites After Pediatric Liver Transplantation Modulates Allograft Rejection Risk
Source: MedComm (2020). 2026 Sep 11;7(9):e71002. doi: 10.1002/mco2.71002 (PMC13570265; doi:10.1002/mco2.71002)
Supplement: Supplementary file 1 [file MCO2-7-e71002-s001.docx]

| **Table S1. Hazard ratio of each clinical parameter to survival after pediatric liver transplantation.** | | | | | | |
| --- | --- | --- | --- | --- | --- | --- |
|  | | | | | | |
| **Variable** | **Total(N)** | **Univariate analysis** | |  | **Multivariate analysis** | |
|  |  | **Hazard Ratio (95% CI)** | **P value** |  | **Hazard Ratio (95% CI)** | **P value** |
| Age at LT, per month | 1,455 | 1.006 (1.000 - 1.012) | 0.052 |  |  |  |
| Primary disease | 1,401 |  |  |  |  |  |
| Cholestatic | 1,111 | Reference |  |  | Reference |  |
| ALF | 24 | 3.686 (1.140 - 11.917) | **0.029** |  | 2.791 (0.824 - 9.457) | 0.099 |
| Metabolic | 143 | 0.604 (0.187 - 1.951) | 0.399 |  | 0.631 (0.192 - 2.077) | 0.449 |
| Tumor | 56 | 3.323 (1.408 - 7.845) | **0.006** |  | 1.750 (0.666 - 4.598) | 0.256 |
| Other | 67 | 2.809 (1.189 - 6.632) | **0.018** |  | 1.192 (0.391 - 3.634) | 0.757 |
| Type of transplantation | 1,455 |  |  |  |  |  |
| SLT | 137 | Reference |  |  | Reference |  |
| LDLT | 1,125 | 0.498 (0.241 - 1.029) | 0.060 |  | 0.512 (0.236 - 1.110) | 0.090 |
| OLT | 193 | 1.089 (0.471 - 2.517) | 0.841 |  | 0.949 (0.390 - 2.309) | 0.907 |
| ABO mismatch | 1,455 |  |  |  |  |  |
| Match | 1,299 | Reference |  |  |  |  |
| Incompatible | 75 | 0.981 (0.307 - 3.134) | 0.974 |  |  |  |
| Compatible | 81 | 0.321 (0.044 - 2.321) | 0.260 |  |  |  |
| GRWR, % | 1,421 | 1.040 (0.822 - 1.317) | 0.741 |  |  |  |
| Recipient CYP3A5 | 1,395 |  |  |  |  |  |
| *1/*3 | 577 | Reference |  |  |  |  |
| *3/*3 | 694 | 1.665 (0.946 - 2.932) | 0.077 |  |  |  |
| *1/*1 | 124 | 1.036 (0.351 - 3.061) | 0.949 |  |  |  |
| Hepatic arterial complication | 1,455 |  |  |  |  |  |
| 0 | 1,445 | Reference |  |  |  |  |
| 1 | 10 | 2.639 (0.366 - 19.044) | 0.336 |  |  |  |
| Biliary complication | 1,455 |  |  |  |  |  |
| 0 | 1,434 | Reference |  |  | Reference |  |
| 1 | 21 | 7.077 (1.728 - 28.981) | **0.007** |  | 0.857 (0.281 - 2.174) | 0.732 |
| Portal venous complication | 1,455 |  |  |  |  |  |
| 0 | 1,442 | Reference |  |  | Reference |  |
| 1 | 13 | 5.172 (1.259 - 21.252) | **0.023** |  | 1.172 (1.087 - 3.231) | **0.042** |
| Blood loss, ml | 1,408 | 1.002 (1.002 - 1.003) | **< 0.001** |  | 1.002 (0.999 - 1.005) | 0.150 |
| RBC infusion, U | 1,304 | 1.363 (1.188 - 1.563) | **< 0.001** |  | 0.947 (0.619 - 1.449) | 0.801 |
| Refractory ascites | 1,455 |  |  |  |  |  |
| No | 921 | Reference |  |  | Reference |  |
| Yes | 534 | 1.764 (1.068 - 2.917) | **0.027** |  | 1.808 (1.098 - 2.976) | **0.020** |
| Acute rejection, episode | 1,455 | 0.568 (0.343 - 0.941) | **0.028** |  | 0.768 (0.448 - 1.316) | 0.337 |
| Chronic rejection | 498 |  |  |  |  |  |
| No | 490 | Reference |  |  |  |  |
| Yes | 8 | 2.481 (0.337 - 18.284) | 0.373 |  |  |  |
| Antibody mediated rejection | 489 |  |  |  |  |  |
| No | 484 | Reference |  |  |  |  |
| Yes | 5 | 0.000 (0.000 - Inf) | 0.997 |  |  |  |

| **Table S2. Hazard ratio of each clinical parameter to graft survival after pediatric liver transplantation.** | | | | | | |
| --- | --- | --- | --- | --- | --- | --- |
|  | | | | | | |
| **Variable** | **Total(N)** | **Univariate analysis** | |  | **Multivariate analysis** | |
|  |  | **Hazard Ratio (95% CI)** | **P value** |  | **Hazard Ratio (95% CI)** | **P value** |
| Age at LT, per month | 1,455 | 1.006 (1.001 - 1.012) | **0.023** |  | 1.007 (0.996 - 1.018) | 0.211 |
| Primary disease | 1,401 |  |  |  |  |  |
| Cholestatic | 1,111 | Reference |  |  | Reference |  |
| ALF | 24 | 3.338 (1.036 - 10.751) | **0.043** |  | 2.131 (0.237 - 19.180) | 0.500 |
| Metabolic | 143 | 1.107 (0.472 - 2.597) | 0.816 |  | 0.601 (0.153 - 2.358) | 0.465 |
| Tumor | 56 | 3.062 (1.304 - 7.192) | **0.010** |  | 2.528 (0.722 - 8.857) | 0.147 |
| Other | 67 | 2.593 (1.104 - 6.090) | **0.029** |  | 0.276 (0.026 - 2.929) | 0.285 |
| Type of transplantation | 1,455 |  |  |  |  |  |
| SLT | 137 | Reference |  |  | Reference |  |
| LDLT | 1,125 | 0.450 (0.232 - 0.874) | **0.018** |  | 0.247 (0.081 - 0.751) | **0.014** |
| OLT | 193 | 1.022 (0.474 - 2.202) | 0.957 |  | 0.949 (0.311 - 2.901) | 0.927 |
| ABO mismatch | 1,455 |  |  |  |  |  |
| Match | 1,299 | Reference |  |  |  |  |
| Incompatible | 75 | 1.529 (0.615 - 3.802) | 0.361 |  |  |  |
| Compatible | 81 | 0.300 (0.042 - 2.163) | 0.232 |  |  |  |
| GRWR, % | 1,421 | 1.044 (0.837 - 1.302) | 0.705 |  |  |  |
| Recipient CYP3A5 | 1,395 |  |  |  |  |  |
| *1/*3 | 577 | Reference |  |  |  |  |
| *3/*3 | 694 | 1.796 (1.042 - 3.094) | **0.035** |  |  |  |
| *1/*1 | 124 | 1.232 (0.460 - 3.300) | 0.678 |  |  |  |
| Hepatic arterial complication | 1,455 |  |  |  |  |  |
| 0 | 1,445 | Reference |  |  |  |  |
| 1 | 10 | 2.639 (0.366 - 19.044) | 0.336 |  |  |  |
| Biliary complication | 1,455 |  |  |  |  |  |
| 0 | 1,434 | Reference |  |  | Reference |  |
| 1 | 21 | 7.077 (1.728 - 28.981) | **0.007** |  | 0.857 (0.281 - 2.174) | 0.732 |
| Portal venous complication | 1,455 |  |  |  |  |  |
| 0 | 1,442 | Reference |  |  |  |  |
| 1 | 13 | 4.838 (1.180 - 19.827) | **0.028** |  |  |  |
| Blood loss, ml | 1,408 | 1.002 (1.001 - 1.003) | **< 0.001** |  | 1.001 (0.997 - 1.005) | 0.656 |
| RBC infusion, U | 1,304 | 1.313 (1.136 - 1.517) | **< 0.001** |  | 1.066 (0.547 - 2.076) | 0.852 |
| Refractory ascites | 1,455 |  |  |  |  |  |
| No | 921 | Reference |  |  | Reference |  |
| Yes | 534 | 1.897 (1.183 - 3.106) | **0.007** |  | 2.321 (1.549 - 3.358) | **0.015** |
| Acute rejection, episode | 1,455 | 0.568 (0.343 - 0.941) | **0.028** |  | 0.768 (0.448 - 1.316) | 0.337 |
| Chronic rejection | 498 |  |  |  |  |  |
| No | 490 | Reference |  |  |  |  |
| Yes | 8 | 2.481 (0.337 - 18.284) | 0.373 |  |  |  |
| Antibody mediated rejection | 489 |  |  |  |  |  |
| No | 484 | Reference |  |  |  |  |
| Yes | 5 | 0.000 (0.000 - Inf) | 0.997 |  |  |  |

**Figure S1**

**Figure S1.** A, RCS plot visualizing the contribution of GRWR to risk for type I (left panel) and type II ascites (right panel); B, DCA curve of predictive models for type I (left panel) and type II ascites (right panel); C, calibration curves and Brier scores of predictive models for type I (left panel) and type II ascites (right panel)
